# Reconstruction of mandibular defects - clinical retrospective research over a 10-year period -

**DOI:** 10.1186/1758-3284-3-23

**Published:** 2011-04-28

**Authors:** Majeed Rana, Riaz Warraich, Horst Kokemüller, Juliane Lemound, Harald Essig, Frank Tavassol, André Eckardt, Nils-Claudius Gellrich

**Affiliations:** 1 Department of Oral and Maxillofacial Surgery, Hannover Medical School, Hannover, Germany; 2 Department of Oral and Maxillofacial Surgery, King Edward Medical University, Lahore, Pakistan

## Abstract

**Backround:**

Functional and cosmetic defects in the maxillofacial region are caused by various ailments and these defects are addressed according to their need. Simplicity of procedure, intact facial function and esthetic outcome with the least possible donor site morbidity are the minimum requirements of a good reconstruction. Oro-mandibular reconstruction, although a challenge for the head and neck reconstructive surgeon, is now reliable and highly successful with excellent long-term functional and aesthetic outcomes with the use of autogenous bone grafts. Reconstruction of trauma- or mandibular oncologic defects with bony free flaps is considered the gold standard. However the the optimal reconstruction of mandibular defects is still controversial in regards to reconstructive options which include the donor site selection and the timing of surgery. The purpose of this study was to determine the outcome of different osseous reconstruction options using autogenous bone grafts for mandibular reconstructions.

**Methods:**

This study was carried out on 178 patients with mandibular bone defects. They were reconstructed with autogenous bone grafts from different donor sites. At post operative visits they were evaluated for functional and cosmetic results.

**Results:**

The success rate found in this study was around 90%. Only 7.6% of the cases showed poor results regarding facial contours and mouth opening. All other patients were satisfied with their cosmesis and mouth opening at the recipient sites was in the normal range during last follow-up visits. Donor sites were primarily closed in all cases and there was no hypertrophic scar.

**Conclusion:**

Based on this study, autogenous bone grafts are a reliable treatment modality for the reconstruction of mandibular bone defects with predictable aesthetic and functional outcomes. As the free vascularized fibular flap has the least resorption and failure rate, it should be the first choice for most cases of mandiblular reconstruction.

## Backround

Functional and cosmetic defects in the maxillofacial region are caused by various ailments that may be congenital, pathologic or iatrogenic such as orofacial clefts, tumor excision and post radiation necrosis [1,2]. Diverse injuries such as motor vehicle accidents, firearms, interpersonal assaults, burns, scalds, electrical flashes and splashes are also playing their part to damage the soft and hard tissue of the whole body in general and the maxillofacial region in particular [3].

Reconstruction of mandibular defects represents a challenge to the head and neck reconstructive surgeon. Interruption of the mandibular continuity produces both a cosmetic and functional deformity. There is limited range of motion when attempting lateral and protrusive movements of the jaw with a return to midline on opening or closing secondary to the remaining contralateral muscles of mastication. In addition, malocclusion and problems with proprioception occur [4,5].

When undertaking mandibular reconstruction, the restoration of bony continuity alone should not be considered the measure of success. The functions of chewing, swallowing, speech articulation and oral competence must also be addressed. The ultimate goal of mandibular reconstruction is to return the patient to their previous state of function. In order to achieve this goal, the reconstructive surgeon must attempt to restore bony continuity and facial contour, maintain tongue mobility and attempt to restore sensation to the denervated areas.

The most common indication for mandibular reconstruction remains ablative surgery for neoplastic processes of the oral cavity and oropharynx. Other causes of mandibular defects include trauma, infection/inflammation, osteoradionecrosis, and congenital deformities. After mandibular resection, particularly following complex radical resection for advanced oropharyngeal carcinomas invading the mandible, the restoration of form and function is paramount for the rehabilitation of these patients [4,6].

Autogenous bone grafting is the mainstay of mandibular reconstruction [7]. Sources of non-vascularized autogenous bone for grafting can be broadly divided into local and distant sites and their successful application to maxillofacial reconstructive surgery is well documented. If the defect requiring a graft is small, often local or intra-oral donor sites are sufficient. When a moderate to substantial amount of bone is required, the distant or extra-oral sites are usually employed [8,9]

Historically, free bone grafts were frequently used for mandibular reconstruction. Autogenous bone grafts from the calvarium, rib, ilium, tibia, fibula, scapula, and radius have been used [10]. Over the past twenty years, however, the use of vascularized bone grafts has become state-of the-art for mandibular reconstruction. The most common donor sites for osseous free-tissue transfer include the fibula, scapula, iliac crest, and radius [7,11]. With the advent of vascularized osseous free flaps over the past thirty years, reliable mandibular reconstruction with success rates of over 90% is possible [11-14].

The field of mandibular reconstruction has seen monumental advances leading to the current state-of-the-art reconstructive techniques. Vascularized osseous free tissue transfer is the preferred reconstructive modality today and has shown excellent long-term aesthetic and functional outcomes. At the present time, autogenous bone grafting is the gold standard by which all techniques of osseous reconstruction of the mandible must be judged and amongst the other available options, is the most reliable and predictable modality to restore the form and function of the lost mandibular segments [5].

## Methods

The aim of this study was to was to determine the outcome of different osseous reconstruction options using autogenous bone grafts for mandibular reconstruction in comparison with the

a) Facial contur and mouth opening

b) Radiodensity of the bone and bone resorption rate

c) Satisfaction and tolerance for the patients

d) Failure of bone grafts

### Subjects

Approval for the study was obtained from the relevant ethics committee. In addition, positive written consent was obtained from each person who participated in the study.

### Patients

The sample consisted of 178 patients. The patients with syndrome like cleft and craniofacial deformities were excluded in the study. Patients with systemic problems, pregnancy, coagulative disorders and drugs were also excluded in the study.

## Results

**Figure 1:**
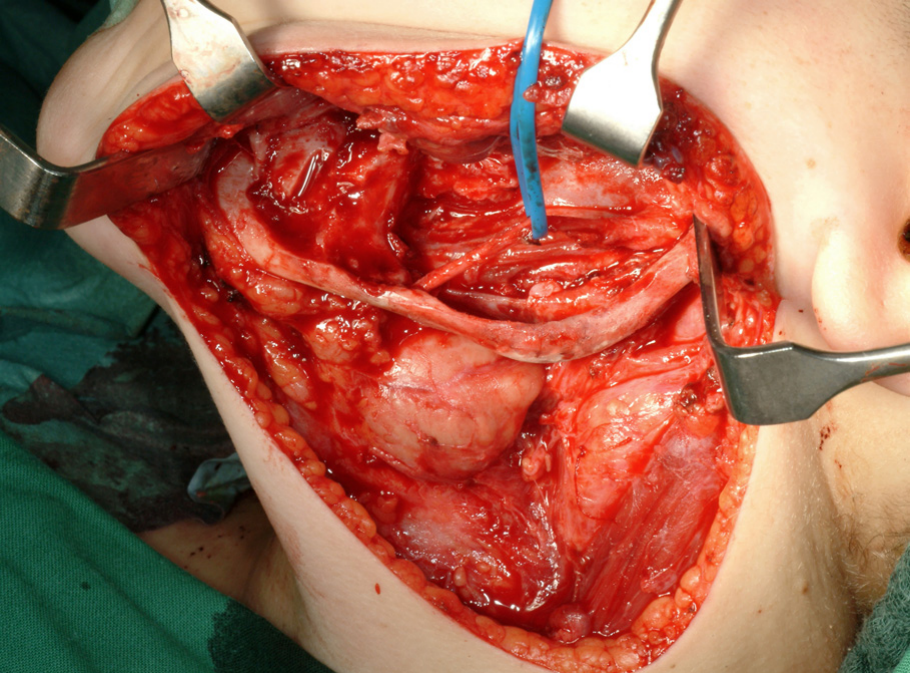
**Left mandibular defect in a 14 year-old boy following removal of a keratocystic odontogenic tumor**. The inferior alveolar nerve was presented as well as mandibular continuity. Primary bone grafting is planned.

The results were collated from 178 patients who reported with mandibular defects and underwent reconstructions with various autogenous bone grafts, in the Department of Oral and Maxillofacial Surgery, from October 1998 to September 2008. All the cases that underwent reconstructive surgery had some defects in the mandible, either primarily or after oncological resection/surgery. There were a total 178 patients, among those 131 (73.6%) patients were males and 47 (26.4%) were females, age ranged from 13-85 years with an average of 55 years at the time of presentation. Among 178 patients, 42.1% (n = 75) patients had oncological resections, 19.6% (n = 35) patients were having post temporomanibular joint ankylosis defects; 24.7% (n = 44) patients had post-traumatic defects and 13.5% (n = 24) patients presented with the ostoeomyelitis of the mandible. Among 75 patients who had defects due to oncological resection, 50 were those presented with squamous cell carcinoma invading the mandible, 05 patients had a keratocystic odontogenic tumor, 09 with Ameloblastoma, 02 patients with Dentigerous cyst, 03 patients with Central Giant Cell Granuloma, 02 with Odontogenic Myxoma, 01 patient with Adenomatoid Odontogenic Tumor, 01 patient with Pindborg tumour and 02 patient had osteosarcoma of the mandible [Table 2], [Figure 1].

**Table 1:** Distribution of Cases by Age and Sex (N = 178)

SEX	NO. OF PATIENTS	PERCENTAGE	MEAN AGE
MALE	131	73.6%	55.1 yrs
FEMALE	47	26.4%	55.4 yrs
**TOTAL**	**178**	**100 %**	

**Table 2:** Distribution of Cases by Etiology of Defects (N = 178)

ETIOLOGY	NO. OF PATIENTS	PERCENTAGE
ONCOLOGICAL RES.	75	42.1%
TMJ ANKYLOSIS	35	19.6%
POST-TRAUMATIC	44	24.7%
OSTEOMYELITIS OF MANDIBLE	24	13.5%
**TOTAL**	**178**	**100 %**

**Figure 2:**
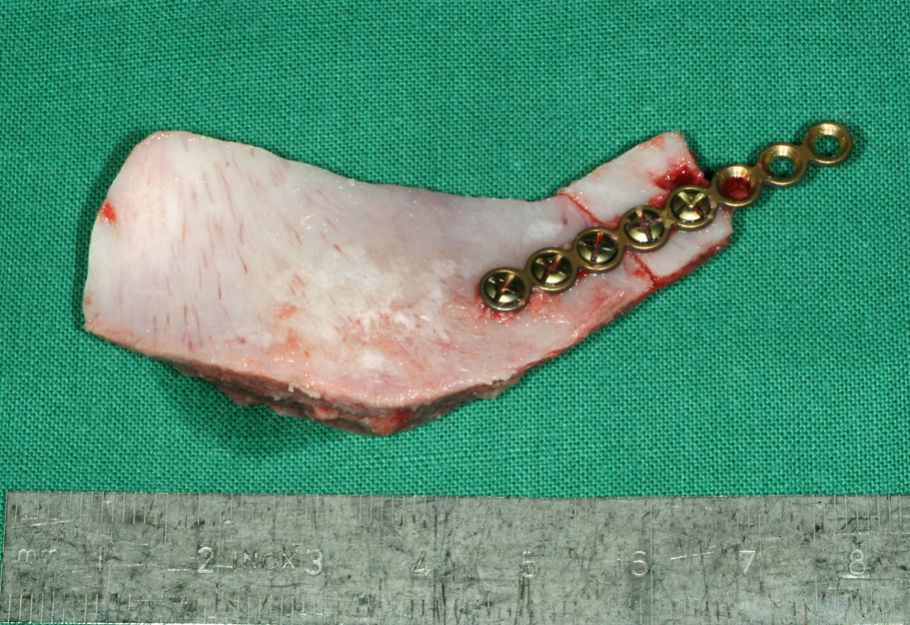
**Contomed monocortical bone graft from the iliac crest for reconstruction of lateral mandibular cortex**.

Among the 44 patients who had post-traumatic defects were 22 patients with infected malunion of fracture sites; 19 patients had the firearm injuries and three patients had the comminuted fracture of the mandible due to road traffic accidents. None of the patients had undergone any previous reconstructive surgical procedure for the mandibular defects [Figure 2].

**Figure 3:**
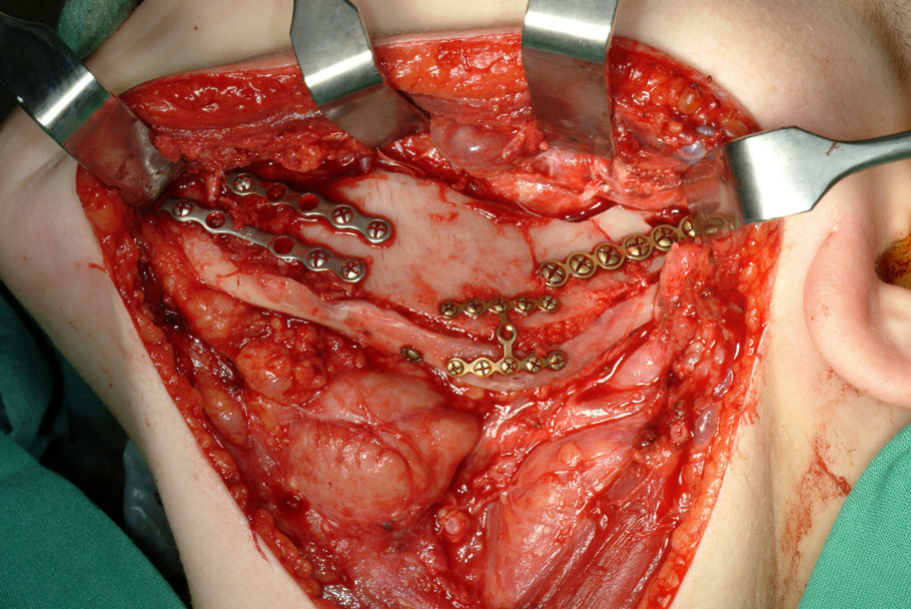
**Bone graft fixation was achieved using 2.0 mm titanium miniplates**. Additional autogenous bone chips were harvested to fill gaps.

All patients who underwent reconstructive surgery, had defects, which affected the patients' normal lives, therefore, they willingly opted for reconstruction with bone grafts [Figure 3].

Among the 178 total patients, 80 (44.9%) had iliac bone grafts, 12 (6.7%) ilium with DCIA, 39 (22.1%) patients with rib grafts were harvested for reconstruction, while 31 (17.4%) patients were reconstructed with the free fibular flap and in 16 (9.1%) patients reconstruction was accomplished with bone graft from the sternum. Of 92 patients of ilium, 80 (44.9%) patients were reconstructed with the iliac bone graft and 12 (6.7%) patients of ilium with DCIA. All these patients experienced mild to moderate pain at donor site which was well managed with analgesics [Table 3].

**Table 3:** Demonstrating the Donor Graft Sites of Patients Receiving Mandibular Reconstruction (N = 178)

DONOR GRAFT SITES	NO OF PATIENTS	PERCENTAGE/%
ILIAC GRAFT	80	44.9%
ILIUM WITH DCIA	12	6.7%
RIB	39	22.1%
FREE FIBULA	31	17.4%
STERNUM	16	9.1%
**TOTAL**	**178**	**100.0%**

Five (5.4%) patients had a postoperative limp, which resolved after a median of 07 days (range 01-25). There were two (2.2%) superficial donor site infections, which resolved well with antibiotic cover. The median length of scar was 60 mm (range 40-90). Only three (3.3%) patients experienced mild paresthesia of the skin supplied by the lateral cutaneous nerve of the thigh, which improved over the time period of six months. No cases of incisional hernia or with permanent gait changes was noted. Overall, harvesting bone from iliac crest was well tolerated by patients with excellent aesthetic and functional results. In 39 (22.1%) patients, rib grafts were harvested for reconstruction and apart from mild to moderate pain, no serious complication was noticed. In one patient, pleural tear occurred which was successfully repaired with uneventful recovery. Bone harvesting from rib proved to be a reliable source with a very few complications. But the resorption rate was much higher than others. Of 31 (17.4%) patients who were reconstructed with the free fibular flap, no major complication was noted, in two cases there were mild wound infection, limited to superficial skin slough, which was managed successfully with the local measures and with antibiotic cover [Table 4].

**Table 4:** Distribution Ofinfections up to 1 Year after Mandible Reconstruction (N = 178)

INFECTION IN BONE GRAFTS	**At 7**^**th **^**day follow-up**		At 1st month follow-up		At 6 months follow-up		At 1 year follow-up	
	
	No. of pts.	%	No. of pts.	%	No. of pts.	%	No. of pts.	%
**PRESENT**	18	10.0	8	4.5	03	1.7	02	1.1
**ABSENT**	160	90	170	95.5	175	98.3	176	99.9
**TOTAL**	**178**	**100.0**	**178**	**100.0**	**178**	**100.0**	**178**	**100.0**

Another patient presented with mild ankle stiffness but that resolved well over the two weeks time period. Overall, reconstruction with the free fibula grafts proved to be very predictable with excellent results. 16 (9.1%) patients received sternum bone graft for reconstruction with pectoralis major muscle as a soft tissue cover. These patients experienced decreased weight lifting capacity after the surgery. One patient presented with suture dehiscence and mild wound infection at the donor site which resolved with the antibiotics and local wound care. No long term donor site morbidity was noted in these patients. Infection was checked postoperatively and was assessed whether present or not. On 7^th ^day postoperatively, 18 (10%) patients developed mild infection and in the rest of 160 (90%) patient's bone grafts, no sign of infection was noted. On 1^st ^month follow up visit, 08 (4.5%) patients developed infection with pus discharge but 170 (95.5%) patients had no sign of infection. On 6^th ^months follow up visits, there was improvement in the infection rate and 03 (1.7%) patients presented with the infection while in the rest of 175 (98.3%) patients, no infection was noted. While on their one year follow up visits, there was further improvement and only 02 (1.1%) patients were noted with infection but in the remaining 176 (99.9%) patients, no signs of infection were noted.

### Facial contour

Facial contour was first recorded preoperatively. Only 10 (5.6%) patients had adequate facial contouring on initial presentation while 168 (94.4%) patients presented with a poor facial profile. After reconstruction with the bone grafts there were marked improvements postoperatively. On one year follow up visits, 140 (79.5%) patients had good facial profiles, 26 (15.1%) patients had adequate results and only 12 (7.6%) patients remained with the poor facial contour [Table 5].

**Table 5:** Post Operative Facial Contour up to 1 Year after Mandible Reconstruction (N = 178)

FACIAL CONTOUR	PRE-TEST		POST-TEST							
	No. of pts	%	**At 7**^**th **^**day follow-up**		At 1st month follow-up		At 6 months follow-up		At 1 year follow-up	
			
			No. of pts.	%	No. of pts.	%	No. of pts.	%	No. Of pts.	%
**GOOD**	00	00	142	80.7	140	79.5	143	80.3	140	79.5
**ADEQUATE**	10	5.6	26	15.1	25	14.0	24	13.5	26	15.1
**POOR**	168	94.4	10	6.2	13	7.3	11	6.2	12	7.6
**TOTAL**	**178**	**100**	**178**	**100**	**178**	**100**	**178**	**100**	**178**	**100**

### Radiodensity of the bone and bone resorption rate

Radiodensity of the bone grafts was checked on radiographs postoperatively and was rated as GOOD, PARTIAL or LUCENT. On 7^th ^post operative day bone grafts of all 178 patients showed GOOD radiodensity, as no changes could occur in bone densities, while in the one year follow up visits, out of 178 patients, radiodensity was GOOD for 147 (82.6%) patient's bone grafts, it was PARTIAL for 17 (9.5%) patients and LUCENT for the bone grafts of 14 (7.9%) patients. Resorption of individual bone grafts was checked only postoperatively on radiographs on the 7^th ^day, 1^st ^month, 6 months and 1 year follow-ups. Out of 178 patients, the highest bone resorption after 1 year was found in rib grafts i.e 64.1% followed by sternum 25%, iliac graft 23.7%, ilium with DCIA 16.7% while the least resorption after one year was found in free fibula grafts i.e 9.1% [Table 6].

**Table 6:** Distribution of Radiodensity after Mandible Reconstruction (Post-test) (N = 178)

RADIODENCITY OF BONE GRAFTS	**At 7**^**th **^**day follow-up**		At 1st month follow-up		At 6 months follow-up		At 1 year follow-up	
	
	No. of pts.	%	No. of pts.	%	No. of pts.	%	No. of pts.	%
**GOOD**	178	100	171	96.1	162	91.0	147	82.6
**PARTIAL**	00	00	03	1.7	09	5.1	17	9.5
**LUCENT**	00	00	04	2.2	07	3.9	14	7.9
**Total**	**178**	**100.0**	**178**	**100.0**	**178**	**100.0**	**178**	**100**

### Failure of bone grafts

Failure of bone grafts was assessed postoperatively only and it was based on the infection, radiodensity and resorption of the harvested bone grafts. Failure was noted whether it occurred or not. No failure of any bone graft reconstruction was noted on the 7^th ^day postoperatively. On the 1^st ^month follow up visit, failure of 07 bone grafts were noted, 16 at the 6 month follow-up while failure of 31 patient's bone grafts were noticed 1 year post operatively. Among 178 patients, failure of bone grafts were noted in 31 (17.4%) patients and the remaining 147 (82.6%) patients, bone grafting was successful on the one year follow up visit [Table 7], [Table 8].

**Table 7:** Results of Resorption of Bone Graft up to 1 Year after Mandible Reconstruction (N = 178)

	RESOPTION OF BONE GRAFTS				
DONOR SITE (total No. of cases)	**7**^**th **^**DAY FOLLOW UP**	**1**^**st **^**MONTH FOLLOW UP**	6 MONTHS FOLLOW UP	1 YEAR FOLLOW UP	TOTAL (%)
Ilium (n = 80)	00	02	07	10	**19 (23.7%)**
Ilium with DCIA (n = 12)	00	00	01	01	**02 (16.7%)**
Rib (n = 39)	00	03	06	16	**25 (64.1%)**
Free Fibula (n = 31)	00	01	01	02	**04 (12%)**
Sternum (n = 16)	00	01	01	02	**04 (25%)**

**Table 8:** Results of Bone Graft Failure after Mandibular Reconstruction (N = 178)

FAILURE OF BONE GRAFTS	**At 7**^**th **^**day follow-up**		At 1st month follow-up		At 6 months follow-up		At 1 year follow-up	
	
	No. of pts.	%	No. of pts.	%	No. of pts.	%	No. of pts.	%
**YES**	00	00	07	3.9	16	9.0	31	17.4%
**NO**	178	100	171	96.1	162	91	147	82.6%
**Total**	**178**	**100.0**	**178**	**100.0**	**178**	**100.0**	**178**	**100**

## Discussion

Reconstruction in the oral and maxillofacial region is a difficult task. Anatomical, functional and aesthetic aspects have to be taken into account while performing reconstructive surgery. Facial contours and animation have to be achieved; normal speech, deglutition and movements of the jaw are to be considered; upper aerodigestive function has to be ensured. Aesthetic units need to be kept in mind and the donor site impairment has to be avoided. Though there are many reconstructive options, from alloplastic bone substitutes to the autogenous bone grafts; the best suited reconstruction option for a particular patient is critical for the restorations of mandibular form and function. A total number of one hundred and seventy eight patients were included in our study. Among them 131 were males and 47 were females and people of various ages were included in this study sample. The same surgeon performed all the procedures to reduce the bias. In oncological resection the tumours affecting the mandible, for example, a squamous cell carcinoma, ameloblastoma, Pindborg tumour, adenomatoid odontogenic tumour, central giant cell granuloma, odontogenic myxoma were included and the cystic lesions like dentigerous cyst, odontogenic keratocyst; while the post-traumatic defects and the cases of osteomyelitis of the mandible were also included in the study sample. The most common indication for the reconstruction in our study was the oncological resection (42.1%) secondary to the benign or malignant diseases of the mandible, followed by post-traumatic defect (24.7%); post-operative defects after gap arthroplasty in TMJ ankylosis (19.6%) and the osteomyelitis of the mandible (13.5%). In a similar study carried out by Szpindor [15], the most common indication for the reconstruction of the mandibular defects was oncological resection, followed by resections due to osteodystrophy, osteoradionecrosis and trauma.

In our study the success rate of reconstruction with autogenous bone grafts was 82.6% (147 patients) while the rate of the graft failure was 17.4% (31 patients). Szpindor [15] demonstrated the positive results or success rate of bone grafts of 84%, though his study sample (n = 64) was smaller than that of ours (n = 178), the success rate in our study, is compareable to Szpindor study. In our study the failure of bone grafts was noted in 17.4 % (n = 31) of patients, while many other factors attributed to the failure of these bone grafts. In two patients the failure of the bone graft was due the recurrence of the squamous cell carcinoma which also got secondarily infected and we had to remove the bone grafts and the mini plates along with the local excision of the recurrent lesion. These patients were subsequently reconstructed with the iliac crest bone graft. While in three patients, there were intraoral extrusion of the bone grafts with associated dehiscence of the sutures and pus discharge. Curettage of the area was undertaken in these patients along with removal of the dead bone to treat the osteomyelitis of the mandible, which was not resolved in spite of giving the antibiotic cover and bone graft from the rib. The contributing factors to the failure of bone grafts in these cases might be the longstanding chronic osteomyelitis of the mandible, additional infection and advanced age of the patient; all these led to poor wound healing with final failure of the bone grafts. In another patient, the failure of the bone graft was noted, who was found to be diabetic after surgery and developed infection with an orocutaneous fistula. This patient also had a history of firearm injury with a comminuted fracture of the mandibular body area and reconstruction was undertaken with a rib graft. Though vigorous debridement of the infected fractured site was done prior to grafting, the underlying immunocompromised host defense with longstanding infection, might have caused failure of the bone graft.

Chiapasco et al [16], in their recently published study on similar lines, described that no total failure of the graft was observed, while partial loss of the graft was observed in one patient. Cumulative survival and success rates were 96.7% and 93.3%, respectively. The success rate is higher to that of our study i.e. 82.6 % (n = 147) and 17.4% (n = 31) failure of the grafts. Underlying comorbidities could be implicated in those patients.

We also noted facial contour/profile restored by the bone grafts in the reconstructed patients. In general, the contour was restored well in all those patients in whom we could maintain the continuity of the mandible compared to those patients with a continuity defect. It's partly because of the fact that correct anatomical position (alignment) of the mandibular resected segments and their deviation after a continuity defect is known to be difficult to achieve.

Preoperatively only 10 patients presented with ADEQUATE and 168 patients were with POOR facial profile while after reconstruction, on one year follow up visits 140 (79.5%) patients were satisfied and had GOOD facial contour. 26 (15.1%) patients had ADEQUATE and 12 (7.6%) patients remained with the POOR facial contours. Among these two who were not satisfied with their poor contour, one was the patient with the osteomyelitis, who also presented with the orocutaneous fistula after reconstruction with rib graft but the infection did not resolve with the local measures and antibiotics. The second patient had a firearm injury of the mandible and rib graft failed to be taken up with formation of a discharge sinus and the facial contour was not up to the mark.

Hidalgo and Pusic [17] reported on the aesthetic outcome in their study and it was excellent to good in 75% of patients, fair in 15% and poor in 10% cases. This difference is not that much higher than that of our study. One reason for achieving more excellent aesthetic outcome is that in Hidalgo DA, Pusic AL's study, most of the reconstructions were accomplished with free microvascular flaps which resulted in more pleasing and predictable outcomes.

Cordeiro PG et al [18], evaluated aesthetic and functional results in their study; they judged the aesthetic outcome as excellent (32%), good (27%), fair (27%) and poor (14%) This study demonstrates a very high success rate, with good to excellent functional and aesthetic results using osseous free flaps for primary mandible reconstruction. They evaluated the patients after six months postoperatively, and we adopted a one year follow up period for our study. Our study shows slightly superior aesthetic results than those but the difference is not significant, as their study sample was smaller (n = 150) than that of ours (n = 178).

Radiodensity of the bone grafts was checked on radiographs postoperatively and was rated as GOOD, PARTIAL or LUCENT. On 1^st ^month follow up visit only 03 bone grafts showed PARTIAL and 04 LUCENT while the remaining 171 patient's bone grafts were rated as GOOD. On the 6 month visit bone grafts of 162 patients showed GOOD, 09 PARTIAL and only 07 bone grafts were rated as LUCENT. In the final one year follow up visit, out of 178 patients, radiodensity was GOOD for 147 (82.6%) patient's bone grafts, it was PARTIAL for 17 (9.5%) patients and LUCENT for the bone grafts of 14 (7.9%) patients only.

Myoung [19] also described almost the same radiodensity rating of the bone grafts in their study. They showed rib as more radiodense than the fibula but the finding was reversed in our study.

We also assessed the resorption of the bone grafts radiograghically on post operative follow up visits om the 7^th ^day, one month, 6^th ^month and 1 year follow-up visits. Out of 178 patients the highest bone resorption after 1 year was found in rib grafts i.e 64.1%, followed by sternum 25%, iliac graft 23.7%, ilium with DCIA 16.7%. The least resorption after one year was found in free fibula i.e 9.1%.

Szpindor [15] demonstrated that in patients with immediate reconstruction, more than 50% of the bone grafts resorbed. Out of 55 bone grafts which showed severe resorption after one year, 16 were rib grafts, 10 iliac crest bone graft, 01 was an iliac crest bone graft with DCIA and 2 were of the sternum. The 02 fibular bone flaps showed moderate resorption but these were taken up successfully. 16 ribs and 11 iliac crest grafts showing severe resorption and the bone grafts were not taken up and ended up in failure. The grafts with moderate and severe resorption also showed partial and lucent radiodensity on radiographs. Lenzen C, Meiss A, Bull HG [20] in their study reported lower resorption with the calvarial bone grafts than when using iliac bone grafting. But we never used calvarial bone graft for mandiblular reconstruction.

## Conclusions

Reconstruction of mandibular defects represents a challenge to the head and neck reconstructive surgeon. Autogenous bone grafting produce the most successful and predictable results when selected from the available reconstruction options for mandibular bone defects. At the present time, autogenous bone grafting is the gold standard by which all techniques of osseous reconstruction of the mandible must be judged, and amongst the other available options, is the most reliable and predictable modality to restore form and function of the missing mandibular segments. The free vascularized fibular flap has the least resorption and failure rate as proven in our study hence it should be the first choice for most cases, particularly those with anterior or large bony defects requiring multiple osteotomies.

## Conflict of interest statement

The authors declare that they have no competing interests.

## Authors' contributions

MR, RW, JL, HE, HK, FT, AE and NCG conceived of the study and participated in its design and coordination. MR drafted the manuscript. All authors read and approved the final manuscript.

## Funding

The article processing charges are funded by the Deutsche Forschungsgemeinschaft (DFG), "Open Acess Publizieren".
